# Cognition, Behavior, and Respiratory Function in Amyotrophic Lateral Sclerosis

**DOI:** 10.5402/2012/912123

**Published:** 2012-07-17

**Authors:** Adriana M. Strutt, Jennette Palcic, John G. Wager, Courtney Titus, Claire MacAdam, Jeffrey Brown, Bonnie M. Scott, Yadollah Harati, Paul E. Schulz, Michele K. York

**Affiliations:** Department of Neurology, An ALS Association Certified Center, Baylor College of Medicine, 6550 Fannin, Suite 1801, Houston, TX 77030, USA

## Abstract

*Objective*. To examine the relationship between respiratory functioning and neuropsychological performance, mood, and frontal-lobe-mediated behaviors in ALS patients. *Methods*. Forty-four patients with probable or definite ALS (El Escorial criteria) completed comprehensive pulmonary and neuropsychological assessments as part of their baseline neurological evaluation. Based on their full vital respiratory capacity, 24 and 20 patients were classified as having impaired or intact respiration, respectively. *Results*. Comparable demographic characteristics, neuropsychological performance, and self-reported mood symptoms were found between ALS patients with intact versus impaired respiration. However, more respiratory-impaired patients were reported by their caregivers as having clinically significant impairments in frontal-lobe-mediated behaviors. Nevertheless, declines in behavior were evidenced from pre- to post-ALS symptom onset for both respiratory groups, and exploratory analyses revealed greater executive functioning deficits in patients with bulbar versus limb onset as well as respiratory-impaired patients not receiving pulmonary interventions versus those utilizing such interventions at the time of testing. *Conclusions*. Results suggest that the respiratory insufficiency of ALS patients may potentially produce irreversible deficits in executive functioning; yet once treated, impairments in more basic cognitive abilities may be less evident.

## 1. Introduction

Although motor system deficits are prominent in amyotrophic lateral sclerosis (ALS), this multisystem disorder is also accompanied by at least subtle cognitive changes in approximately 40–50% of patients [1–3]. The clinically significant declines observed in these patients encompass a variety of cognitive skills including attention, verbal fluency, short-term memory, visuospatial abilities, and executive functioning, revealing a frontotemporal pattern of dysfunction [1, 2, 4–8]. However, predictors for such alterations in mental faculties have not been identified [9].

Sleep-disordered breathing (SDB) [10] afflicts a considerable proportion of patients with ALS—especially those with bulbar involvement and executive dysfunction—and is one of the principal deficits observed in individuals with SDB [11–14]. Respiratory insufficiency, hypoxemia, and hypocarbia resulting from the muscle weakness characteristic of the disease process have been proposed as one of the causal mechanisms underlying the aforementioned cognitive declines in ALS; however, a consensus regarding this hypothesis has yet to be reached given inconsistent findings in this area of research [12, 15–17]. Therefore, the purpose of the present study was to examine the relationship between respiratory functioning and neuropsychological performance, mood, and frontal-lobe-mediated behaviors in a cohort of patients diagnosed with ALS. 

## 2. Materials and Methods 

### 2.1. Participants

Forty-four patients from Baylor College of Medicine's (BCM) ALS clinic who met probable or definite diagnostic criteria according to the El Escorial World Federation of Neurology [18] underwent comprehensive pulmonary and neuropsychological assessments as part of their baseline neurological evaluation. Patients were classified as having impaired or intact respiration based on their full vital respiratory capacity (FVC). Twenty-four ALS patients with FVC percentages of ≤80% were classified as having impaired respiration and were compared to their nonimpaired respiration counterparts (*n* = 20). Half of those participants classified as having impaired respiration were receiving pulmonary interventions at the time of testing including 1 nocturnal ventilator, 1 C-PAP, and 10 Bi-PAP. Exclusion criteria included (1) estimated IQ score <70, (2) current or past psychotic symptoms or chronic mental health disorders that could impact diagnostic assessment, (3) substance abuse disorder (per DSM-IV TR) within the past 6 months, (4) treatment with an experimental drug or device within 60 days of study enrollment, and/or (5) history of head trauma. All participants provided informed consent, and the research protocol was approved by BCM's Institutional Review Board. 

### 2.2. Measures

Demographic data was obtained via individual clinical interviews. Subsequently, each participant was administered a comprehensive neuropsychological assessment that examined basic orientation [Mini-Mental State Examination (MMSE)], [19] attention/information processing speed [Digit Span subtest of the Wechsler Adult Intelligence Scale-Third Edition (WAIS-III), Trail Making Test, part A (Trails A), and Verbal Sustained Attention Test (VSAT)], [20–22] verbal and visual learning [Rey Auditory Verbal Learning Test (RAVLT) and Brief Visual Memory Test-Revised (BVMT-R)], [23, 24] language [Boston Naming Test (BNT) and semantic fluency (Animals)], [25, 26] visuo-spatial abilities [Rey-Osterrieth Complex Figure Test (Rey-O) and Block Design subtest of the WAIS-III], [20, 27] executive function [Wisconsin Card Sorting Test (WCST), Similarities subtest of the WAIS-III, Trail Making Test-part B (Trails B), and lexical fluency (FAS)], [20, 21, 26, 28] mood [Beck Depression Scale-Second Edition (BDI-II) and Beck Anxiety Inventory (BAI)], [29, 30] and behavior [self-rating form of the Frontal Systems Behavior Scale (FrSBe)] [31].

Additionally, severity of physical impairments associated with their diagnosis of ALS was examined [Amyotrophic Lateral Sclerosis Functional Rating Scale-Revised (ALSFRS-R)], [32] and the caregiver who accompanied each patient was asked to rate the patient's frontal-lobe-mediated behavioral tendencies, particularly apathy, disinhibition, and executive dysfunction using the family-rating form of the Frontal Systems Behavior Scale (FrSBe) [31]. As *t*-scores ≥65 on this measure are considered indicative of clinically significant declines in frontal-lobe-mediated behaviors, this cutoff was employed in analyses between respiratory groups [31]. The sample size varied between the neuropsychological measures, mood questionnaires, and the behavioral inventories due to the physical limitations (e.g., fatigue, limited motor abilities) or time constraints of the patient. 

### 2.3. Statistical Analyses

All statistical analyses were conducted via IBM SPSS version 19.0. *T*-tests and chi-squares were calculated to examine differences on neuropsychological performance, mood, and behavioral tendencies between those with impaired and intact respiratory functioning, ALS patients with bulbar versus limb onset, and respiratory impaired patients receiving and not receiving pulmonary interventions at the time of testing. Repeated measures ANOVAs were used to examine the main effects and interaction of frontal-lobe-mediated behavioral changes over time (from presymptomatic condition to current status) and the possibility of an interaction between behavioral changes over time (from pre-ALS symptomatology to current status) and group (impaired versus intact respiration) on both the family and the self-rating forms of the FrSBe. 

## 3. Results

A comparison of the demographic variables and neuropsychological outcome measures between intact and impaired respiratory groups is provided in Tables 1 and 2, respectively. As shown, no significant between-group differences were found on demographic variables, including age, education, gender, ethnicity, site of disease onset, and physical impairments associated with ALS as reported on the ALSFRS-R. Furthermore, no significant differences were evidenced between those with impaired and nonimpaired respiration on tests of basic orientation, auditory attention, working memory, information processing speed, verbal and visual memory, language, visuo-spatial abilities, and higher-order cognitive functioning. Moreover, chi-square analyses indicated that the number of ALS patients with moderate cognitive impairment, defined by Strong et al. [1] as a decline of ≥1.5 standard deviations on two or more neuropsychological domains (including a test of executive functioning), also did not differ significantly between those with impaired (33.3%) versus intact (25.0%) respiration. 

Additionally, no significant differences were observed between respiratory groups in self-reported symptoms of depression or anxiety, nor in pre-ALS onset or current FrSBe behaviors reported by caregivers, including apathy, disinhibition, and executive dysfunction. Furthermore, no significant between-group differences in pre-ALS onset or current frontal-lobe-mediated behaviors were found in the subsample of 19 patients who completed the self-rating form of the FrSBe, and these scores did not differ significantly for either respiratory group from those reported by caregivers on the family-rating form of this measure. For details regarding mood and behavioral tendencies reported for both intact and impaired respiratory groups, see Table 3. 

**Figure 1:**
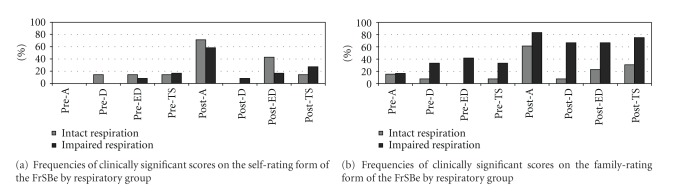
Abbreviations are as follows: A: apathy; D: disinhibition; ED: executive dysfunction; TS: total score.

Utilizing the recommended cut-off score of *T* ≥ 65, [31] no significant differences in frequencies of impairment were found between respiratory groups for the subsample of patients completing the self-rating form of the FrSBe. However, significantly more respiratory-impaired patients were described by their caregivers as having clinically significant impairments in executive functioning before (*χ* ^2^ = 4.31, *P* = 0.04; intact respiration = 0.00%; impaired respiration = 41.7%) and greater disinhibition after (*χ* ^2^ = 4.95, *P* = 0.03; intact respiration = 7.70%; impaired respiration = 66.7%) ALS onset. The frequencies of clinically significant behavioral ratings (i.e., *T* ≥ 65) on the self- and family-rating forms of the FrSBe are depicted for both respiratory groups in Figures 1(a) and 1(b).

When evaluating changes in frontal-lobe-mediated behaviors from pre-ALS symptom onset to current symptoms reported on the family-rating form of the FrSBe, a significant time main effect was found, with ALS caregivers reporting an increase in apathy (*F*(1,31) = 26.0, *P* < 0.001), disinhibition (*F*(1,31) = 6.95, *P* = 0.01), executive dysfunction (*F*(1,31) = 8.01, *P* = 0.008), and total behavior scores (*F*(1,31) = 16.6, *P* < 0.001) following the onset of ALS for all patients. However, no significant interaction of time by respiratory group for behavioral changes was observed (apathy: *P* = 0.73; disinhibition: *P* = 0.32; executive dysfunction: *P* = 0.57; total behavior score: *P* = 0.93) as both groups endorsed similar behaviors. Comparable results were also found in the subsample of 19 patients who completed the self-rating form of the FrSBe. That is, significant increases in apathy (*F*(1,17) = 28.3, *P* < 0.001), disinhibition (*F*(1,17) = 4.38, *P* = 0.05), executive dysfunction (*F*(1,17) = 8.25, *P* = 0.01), and total behavior scores (*F*(1,17) = 23.9, *P* < 0.001) were found from pre-ALS symptomatology to current status. Also commensurate with the results found on the FrSBe family-rating form, no significant interaction between a change in behavioral tendencies by respiratory group was observed (apathy: *P* = 0.35; disinhibition: *P* = 0.99; executive dysfunction: *P* = 0.42; total behavior score: *P* = 0.37).

Secondary analyses revealed no significant demographic differences between ALS patients with bulbar versus limb onset. However, a significant difference was found between these two groups on trails B (*t*(40) = 3.08, *P* = 0.004), as well as on the FrSBe executive dysfunction subscale (*t*(17) = 2.21, *P* = 0.04) and total behavior score (*t*(17) = 2.22, *P* = 0.04) before ALS onset and disinhibition (*t*(17) = 3.91, *P* = 0.001) reported after ALS onset by patients on the self form of the FrSBe—with bulbar onset patients reporting greater impairments in frontal-lobe-mediated behaviors and demonstrating greater executive functioning deficits than their limb-onset counterparts. Additionally, significant differences on neuropsychological executive functioning measures were observed between respiratory-impaired patients receiving (*n* = 12) and not receiving (*n* = 12) pulmonary interventions (WAIS-III Similarities: *t*(20) = −2.36, *P* = 0.03; WCST categories: *t*(19) = −2.57, *P* = 0.02), as those patients undergoing respiratory interventions obtained better scores on both measures. However, no significant between-group differences were observed on any other neuropsychological measure, nor were significant differences in frontal-lobe-mediated behaviors reported between these intervention groups by either patients or their caregivers on the FrSBe, and FVC scores were not significantly correlated with any of the outcome measures.

## 4. Discussion

The present study examined the relationship between respiratory functioning and neuropsychological performance, mood, and frontal-lobe-mediated behaviors in a cohort of patients diagnosed with ALS. Results revealed comparable demographic characteristics, neuropsychological performance, and self-reported symptoms of anxiety and depression between ALS patients with intact versus impaired respiration. Additionally, while the raw scores of self and family ratings on the FrSBe were also comparable between respiratory groups, significantly more respiratory-impaired patients were reported by their caregivers as having clinically significant impairments in executive functioning before and greater disinhibition after ALS onset. More patients with intact respiration reported clinically significant scores on the self-rating form of the FrSBe, while a larger number of respiratory-impaired patients were described by their caregivers as having clinically significant scores on the family-rating form of the FrSBe. Nevertheless, a change in apathy, disinhibition, executive dysfunction, and total behavior scores was noted by patients as well as caregivers for both respiratory groups from pre-ALS symptom onset to the patients' current status. Subsequent exploratory analyses also revealed greater executive dysfunction in patients with bulbar versus limb onset, as well as respiratory-impaired patients not receiving pulmonary interventions versus those utilizing interventions at the time of testing. 

More specifically, bulbar onset patients reported greater impairments in executive functioning before ALS and greater disinhibition after ALS symptom onset. Bulbar onset patients demonstrated greater executive dysfunction in comparison to their limb onset counterparts on only one of five indices of executive functioning, and their worse performance was evident on a measure of high-order processing that includes a motor component (i.e., Trails B). Additionally, respiratory-impaired patients undergoing pulmonary interventions at the time of testing obtained better scores than the remaining untreated respiratory-impaired group on two motor-independent measures of executive functioning. Despite such findings, neither respiratory-impaired patients receiving pulmonary interventions nor their caregivers reported better frontal-lobe-mediated behaviors than those not receiving interventions. Furthermore, no significant relation was found between FVC scores and any of the outcome measures.

Previous investigators [10] have suggested that the intermittent blood gas abnormalities, including hypoxemia and hypocarbia, resulting from respiratory insufficiency as that found in ALS patients may potentially produce neurological damage (especially in prefrontal regions of the brain) which is only partially reversible. Such damage is manifested as deficits in executive functioning, which can subsequently impair the recruitment of more basic cognitive abilities (e.g., verbal and visual learning). Despite improvements in these more primary functions reported with successful treatment interventions, residual deficits in executive functioning may continue to be observed [10, 33–35]. Results of the current study are consistent with such findings, as no significant differences were observed between respiratory groups in any cognitive domain; however, ALS patients receiving pulmonary interventions performed better on two measures of executive functioning than those patients who were not. Hence, our results support previous research [10] in that the respiratory insufficiency of ALS patients may potentially produce irreversible deficits in executive functioning; however, once treated, impairments in more basic cognitive abilities (i.e., core language, visuoperception, and rote memory capacity) may be less evident. 

Despite improving previous research by utilizing a larger sample, the main limitation of the current study was the small sample size in conjunction with the high number of statistical analyses employed. Moreover, the pulmonary assessments of ALS patients in the current study did not include the measurement of CO_2_ levels, thereby preventing a direct examination of the relationship between hypocarbia severity and neuropsychological functioning. Further research should explore such relationships in a larger sample with a comprehensive neuropsychological battery and a longitudinal research design, with the addition of reliable changes indices when examining cognitive performance to ensure that noted changes over time are appropriately quantified, accounting for both practice effects and measurement reliability. Additionally, level of physical impairment varied significantly within our sample, and thus, neuropsychological data was not available for the whole sample. As such, the current findings may have been influenced by these varying data points. Finally, the potential influence of disease duration could not be examined as this data was not collected at the time of testing. Future research should consider this variable, and if necessary, control for its influence on neuropsychological performance.

 In sum, the results of the current study suggest that ALS patients with intact versus impaired respiration (as defined by FVC percentages of ≤80%) are demographically similar and possess comparable neurocognitive abilities. However, despite similar performances on neurocognitive measures of executive functioning, more respiratory-impaired patients were reported by their caregivers as having clinically significant impairments in frontal-lobe-mediated behaviors, yet such behavioral declines were evidenced from pre- to post-ALS symptom onset for both respiratory groups. Additionally, exploratory analyses revealed greater executive functioning deficits in respiratory-impaired patients not receiving pulmonary interventions versus those utilizing such interventions at the time of testing, and therefore, current findings suggest that the respiratory insufficiency of ALS patients may potentially produce irreversible deficits in executive functioning, yet once treated, impairments in more basic cognitive abilities may be less evident suggesting that implementation of respiratory interventions in the earlier stages of the illness may be beneficial. 

## Figures and Tables

**Table 1:** Comparison of demographic variables between respiratory groups.

Variable	Intact respiration	Impaired respiration	*P* value
Age (yrs)	55.9 (11.9)	59.7 (11.1)	0.28
Education (yrs)	14.6 (2.62)	14.2 (2.12)	0.55
ALSFRS-R total score	41.7 (5.24)	37.8 (8.22)	0.12
Gender (% Male)	11 (55.0%)	11 (45.8%)	0.55
Race (%)			
Caucasian	17 (85.0%)	20 (83.3%)	0.67
Hispanic	1 (5.00%)	2 (8.30%)	
African American	2 (10.0%)	1 (4.20%)	
Other	—	1 (4.20%)	
Site of onset (%)			
Bulbar	6 (30.0%)	8 (33.3%)	0.81
Limb	14 (70.0%)	16 (66.7%)	

Note: mean (SD) and frequencies (%) are given for each variable. *t*-tests and chi-squares were calculated for continuous (age, education, and ALS-FRS-R) and categorical variables (gender, race, and site of onset), respectively. Abbreviations are as follows: ALSFRS-R: amyotrophic lateral sclerosis functional rating scale-revised.

**Table 2:** Comparison of respiratory groups' neuropsychological performance by cognitive domain.

Measure	Intact respiration	Impaired respiration	*P* value
Basic orientation			
MMSE (1–10)	9.50 (0.86)	9.81 (0.50)	0.15
Working memory and processing speed			
VSAT time	91.2 (44.3)	123 (68.2)	0.10
VSAT errors	2.83 (3.11)	3.20 (5.03)	0.79
WAIS-III Digit Span	15.9 (3.53)	16.7 (4.17)	0.55
Trail Making Test, part A	41.1 (22.5)	43.8 (26.3)	0.72
Verbal memory			
RAVLT total recall	49.2 (11.1)	46.8 (8.89)	0.45
RAVLT immediate recall	10.6 (3.58)	8.85 (3.31)	0.13
RAVLT delay recall	10.6 (3.02)	9.30 (3.54)	0.22
Visual memory			
BVMT-R total recall	19.5 (7.93)	18.6 (7.56)	0.72
BVMT-R delay recall	7.50 (3.62)	7.41 (3.07)	0.93
Language			
Semantic fluency (Animals)	18.4 (6.73)	16.4 (4.60)	0.31
Confrontational naming	54.0 (6.82)	55.1 (3.55)	0.61
Visuoconstruction/spatial			
Rey-O	30.5 (7.02)	33.0 (8.35)	0.85
WAIS-III Block Design	33.8 (13.5)	33.2 (8.94)	0.89
Executive functioning			
WAIS-III Similarities	22.5 (6.61)	22.3 (6.39)	0.91
Trail Making Test, part B	118 (92.6)	124 (78.6)	0.81
Lexical fluency (FAS)	33.7 (14.8)	30.2 (10.3)	0.43
WCST Categories	3.00 (1.83)	3.14 (1.24)	0.78
WCST Perseverative Errors	13.6 (16.3)	11.6 (8.93)	0.64

Note: mean (SD) is given for each variable. Abbreviations are as follows: MMSE: Mini Mental State Examination; VSAT: Verbal Sustained Attention Test; WAIS-III: Wechsler Adult Intelligence Scale-Third Edition; RAVLT:~Rey~Auditory Verbal Learning Test; BVMT-R: Brief Visual Memory Test-Revised; Rey-O: Rey-Osterrieth Complex Figure Test; WCST: Wisconsin Card Sorting Test.

**Table 3:** Mood and behavioral tendencies by respiratory group.

Measure	Intact respiration	Impaired respiration	*P* value
Mood			
BDI-II (0-63)	12.2 (6.31)	11.2 (5.32)	0.65
BAI (0-63)	10.9 (6.56)	13.4 (11.8)	0.54
FRSBE-family: before ALS			
Apathy	22.7 (5.81)	21.9 (6.01)	0.71
Disinhibition	21.2 (5.29)	20.4 (6.52)	0.70
Executive dysfunction	28.0 (4.49)	28.2 (9.53)	0.96
Total score	71.9 (12.2)	70.4 (20.1)	0.81
FRSBE-family: current status			
Apathy	31.4 (8.48)	31.8 (13.5)	0.92
Disinhibition	23.0 (7.53)	24.4 (8.49)	0.64
Executive dysfunction	33.9 (13.9)	32.1 (12.1)	0.70
Total score	89.1 (27.6)	88.3 (32.3)	0.95

Note: mean (SD) is given for each variable. Abbreviations are as follows: BDI-II: Beck Depression Inventory-Second Edition; BAI: Beck anxiety inventory; FRSBE-family: frontal systems behavior scale-family-rating form.
